# Cell Surface and Functional Features of Cortical Bone Stem Cells

**DOI:** 10.3390/ijms222111849

**Published:** 2021-10-31

**Authors:** Norihiko Sasaki, Yoko Itakura, Sadia Mohsin, Tomoaki Ishigami, Hajime Kubo, Yumi Chiba

**Affiliations:** 1 Research Team for Geriatric Medicine (Vascular Medicine), Tokyo Metropolitan Institute of Gerontology, Tokyo 173-0015, Japan; sasanori@tmig.or.jp (N.S.); yitakura@tmig.or.jp (Y.I.); 2 Cardiovascular Research Center, Lewis Katz School of Medicine, Temple University, Medical Education and Research Building, 3500N. Broad St., Philadelphia, PA 19140, USA; sadia.mohsin@temple.edu (S.M.); hajime.kubo@temple.edu (H.K.); 3 School of Medicine, Medical Course, Medical Sciences and Cardiorenal Medicine, Yokohama City University, Yokohama 236-0004, Japan; tommmish@yokohama-cu.ac.jp; 4 Cancer/Advanced Adult Nursing, Department of Nursing, Graduate School of Medicine, Yokohama City University, Yokohama 236-0004, Japan

**Keywords:** cortical bone stem cells, stemness, glycan profile, TGF-β1

## Abstract

The newly established mouse cortical-bone-derived stem cells (mCBSCs) are unique stem cells compared to mouse mesenchymal stem cells (mMSCs). The mCBSC-treated hearts after myocardial infarction have been reported to have greater improvement in myocardial structure and functions. In this study, we examined the stemness features, cell surface glycan profiles, and paracrine functions of mCBSCs compared with mMSCs. The stemness analysis revealed that the self-renewing capacity of mCBSCs was greater than mMSCs; however, the differentiation capacity of mCBSCs was limited to the chondrogenic lineage among three types of cells (adipocyte, osteoblast, chondrocyte). The cell surface glycan profiles by lectin array analysis revealed that α2-6sialic acid is expressed at very low levels on the cell surface of mCBSCs compared with that on mMSCs. In contrast, the lactosamine (Galβ1-4GlcNAc) structure, poly lactosamine- or poly *N*-acetylglucosamine structure, and α2-3sialic acid on both *N*- and *O*-glycans were more highly expressed in mCBSCs. Moreover, we found that mCBSCs secrete a greater amount of TGF-β1 compared to mMSCs, and that the TGF-β1 contributed to the self-migration of mCBSCs and activation of fibroblasts. Together, these results suggest that unique characteristics in mCBSCs compared to mMSCs may lead to advanced utility of mCBSCs for cardiac and noncardiac repair.

## 1. Introduction

Several cardiovascular diseases (CVDs) are the leading cause of death globally due to their high morbidity and mortality rates [1]. In the coming decades, the incidence of CVD caused by ischemic CVDs, such as myocardial infarction (MI), is expected to be in upward trend [2]. After MI, myocyte death and the reduction in the number of functional cardiac myocytes ultimately leads to heart failure. Until now, although there is scientific progress and advancements in surgical techniques, drugs and surgical treatments can only delay the progression of chronic heart disease, but not improve the function of infarcted myocardial cells [3]. Therefore, the use of stem cells has emerged as a promising treatment for heart disease [4]. Our research and others suggest that stem cells hold immense potential for cardiac repair and regeneration [5,6,7,8]. Clinical use of adult somatic stem cells (SSCs) is a reality today and many stem cell types, including bone-marrow-derived mesenchymal stem cells (MSCs) [9], bone marrow cells [10,11], cardiac-derived cardiac progenitor [12], and cardio-sphere-derived cells [13], have been tested. The beneficial effects of tested cell therapies on cardiac structure and function have been modest, and most studies to date have not been adequately powered to document efficacy. The emerging consensus from these studies suggests that the donated stem cell population falls short of fully restoring normal cardiac functional capacity because of a combination of issues, such as poor survival, lack of proliferation, engraftment, and differentiation. In addition, it seems that much of the benefit derived from cell therapy has come from the release of paracrine factors acting on the host myocardium rather than from differentiation of infused/injected stem cells into new cardiac tissue.

Recently, we have shown that a novel SSC, mouse cortical-bone-derived stem cells (mCBSCs), improve cardiac remodeling and functions. The mCBSC-treated hearts showed increased neovascularization, and newly formed cardiac myocytes were also observed [8,14]. mCBSCs produce a unique combination of immunomodulatory and angiogenic and proangiogenic factors, which may be the reason why mCBSCs were more effective in improving the post-MI hearts compared to cardiac-derived stem cells and MSCs [14]. Furthermore, mCBSCs possess enhanced proliferation capacity, better survival, and lineage commitment capacity than those in other stem cells [14]. Thus, it is suggested that CBSCs have greater potential to repair the damaged heart than other cells currently being tested clinically. However, there are still many unknown characteristics in mCBSCs, including stem-cell-like features, noncardiac therapeutic potentials, and cell surface markers. Such characterization is considered to be useful for enhancing the utility of CBSC.

Glycans are expressed mainly on the surface of cells as components of glycoproteins, glycolipids, and proteoglycans, and are used as cell surface markers [15,16]. Glycoproteins contain *N*-glycans (including three major types: high mannose, hybrid, and complex glycans) synthesized on asparagine residues, part of the Asn-X-Ser/Thr consensus sequence (X: any amino acid except proline), and *O*-glycans synthesized on serine or threonine residues. It is well documented that glycan compositions of glycoproteins largely change during stem cell differentiation [17,18,19,20]. Furthermore, cell surface glycans contribute to fundamental biological functions, such as cell differentiation, cell adhesion, cell–cell interactions, pathogen–host recognition, toxin–receptor interactions, cancer metastasis, immune responses, and regulation of signaling pathways [16]. The glycans, including lactosamine and fucosylation, have been implicated in the self-renewal of stem cells by preventing differentiation [19,21,22]. In human MSCs (hMSCs), both *N*- and *O*-glycan processing functionally modulates early steps of osteogenic differentiation [23]. In adipose-derived hMSCs, the ability to differentiate is downregulated, with a decrease in α2–6-linked sialic acid associated with long-term cell culture in so-called “in vitro cellular aging” [24]. Thus, SSCs, including hMSCs, undergo tremendous changes, such as loss of differentiation potential, accompanying changes in glycans during long-term cell culture [25,26,27].

The aim of the present study is to characterize mCBSCs. In this study, we focused on elucidating mCBSC stemness features, therapeutic potentials, and cell surface glycans, including the effect of in vitro cellular aging. In addition, bone-marrow-derived mouse MSCs (mMSCs) were used for comparison. 

## 2. Results

### 2.1. Isolation and Culture of mCBSCs

**Figure 1:**
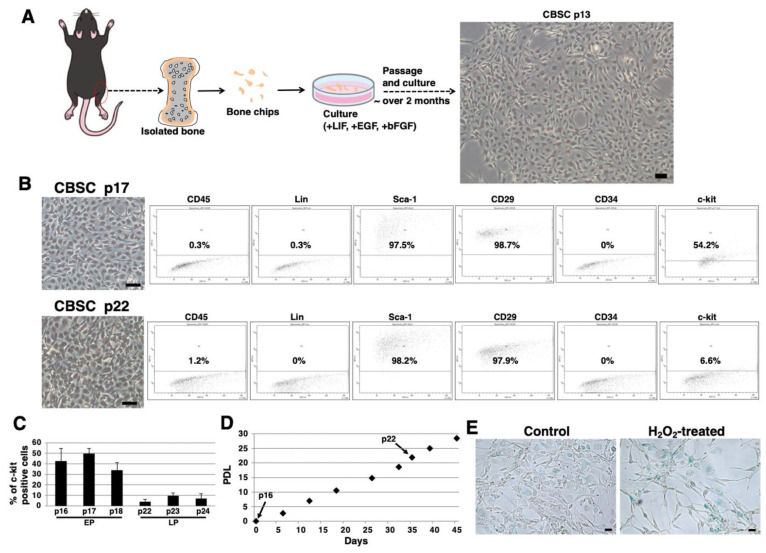
Isolation of mCBSCs. (**A**) Scheme of isolation and culture of mCBSCs. The bone biopsy was isolated from one limb. Then, bone chips were prepared and cultured with mCBSCs culture media. About 2 months after passage and culture, mCBSCs was observed. Scale bar in the phase contrast image: 2 μm. (**B**) FACS analysis of CD45, Lin, Sca-1, CD29, CD34, and c-kit in mCBSCs (p17 and p22). Three independent experiments were performed and representative results are shown. Scale bar in the phase contrast image: 2 µm. (**C**) Percent of c-kit positive cells in mCBSCs (p16-p18 and p22-p24) is shown. The values shown are the means ± SD from three independent experiments. (**D**) Growth curve showing the population doubling level (PDL) during culturing from p16 to p24. (**E**) Representative image of staining for SA-β-Gal in nontreated (control) or 400 µM hydrogen peroxide (H_2_O_2_)-treated LP-mCBSCs from two independent experiments is shown. Scale bar in the phase contrast image: 50 µm. Abbreviations: mCBSC, mouse cortical-bone-derived stem cell; EP, early passage; LP, late passage; PDL, population doubling level; SA-β-Gal, senescence-associated β-galactosidase; H_2_O_2_, hydrogen peroxide.

We isolated mCBSCs with modified protocols, as described in the Materials and Methods section. With modified protocols, mCBSCs were observed after approximately 2 months (passage 13) and cultured (Figure 1A). Fluorescence-activated cell sorting (FACS) analysis showed that the majority of mCBSCs (passage 17) expressed Sca-1 and β1-integrin (CD29), and around 50% of mCBSCs expressed c-kit (CD117) (Figure 1B). In contrast, later passaged mCBSCs (passage 22) showed weaker expression of c-kit, while Sca-1 and CD29 were expressed in the majority of the cells (Figure 1B). In both passaged mCBSCs (passage 17 and 22), CD45, Lin (hematopoietic lineage), and CD34 were not expressed (Figure 1B). We found that around 50% of mCBSCs (passages 16-18; defined early passage, EP) expressed c-kit, while c-kit was expressed in weak populations of mCBSCs (passages 22-24; defined late passage, LP) (Figure 1C). The growth curve showed the expected pattern of rapid growth for about 45 days, including EP and LP (Figure 1D). Furthermore, senescence-associated β-galactosidase (SA-β-Gal) activity, which is a well-known marker for senescent cells, was almost always negative in LP-mCBSCs compared to hydrogen peroxide (H_2_O_2_)-treated LP-mCBSCs, in which stress-induced premature senescence was considered to be induced (Figure 1E). These results indicate that populations of mCBSCs expressing c-kit change during long-term culture in so-called in vitro cellular aging, without cellular senescence. We compared characteristics of mCBSCs (EP and LP) with bone-marrow-derived mMSCs in further experiments.

### 2.2. Stem-Cell-Like Features of mCBSCs

**Figure 2:**
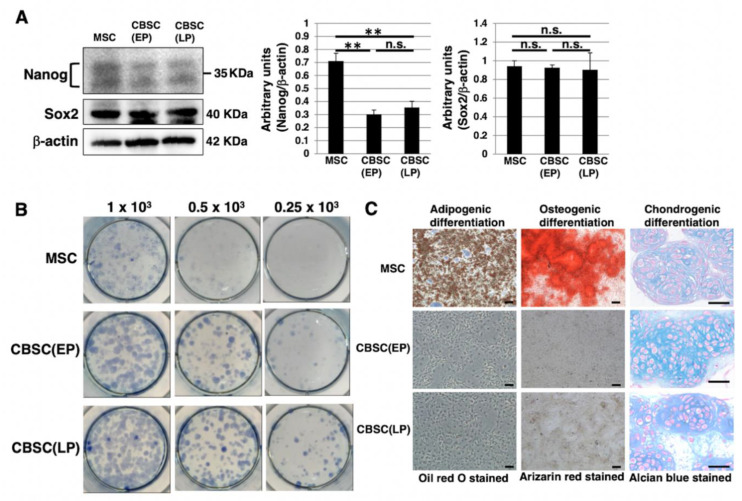
Stemness features of mCBSCs. (**A**) Western blot analysis of Nanog, Sox2, and β-actin (loading control) in mMSCs and mCBSCs (EP and LP). The blot images were cropped to highlight the Nanog, Sox2, and β-actin bands. The histogram shows the mean densitometric analysis ± SD of Nanog or Sox2 normalized to the loading control (β-actin). The values were obtained from three independent experiments. ** *p* < 0.01, n.s.: not significant. (**B**) Colony-forming assay in mMSCs and mCBSCs (EP and LP). Cells were plated at low density (0.25 × 10^3^ cells, 0.5 × 10^3^ cells, or 1 × 10^3^ cells/well) and cultured. After 7 days, cells were stained and photos were captured. Two independent experiments were performed and representative images are shown. (**C**) Adipogenic, osteogenic, or chondrogenic differentiation was induced in mMSCs and mCBSCs (EP and LP). Two independent experiments were performed and representative images are shown. Scale bar in the phase contrast image of adipogenic and osteogenic: 2 µm. Scale bar in the phase contrast image of chondrogenic: 50 µm. Abbreviations: mMSC, mouse mesenchymal stem cell; mCBSC, mouse cortical-bone-derived stem cell; EP, early passage; LP, late passage.

We examined stem-cell-like features of mCBSCs, specifically, Nanog and Sox2, as they are well known as stemness markers in the undifferentiated state of mouse ESCs [28]. In mCBSCs, Nanog was expressed, but lower than that in mMSCs (Figure 2A). In contrast, Sox2 was highly expressed in mCBSCs, comparable to that in mMSCs (Figure 2A). We further examined stemness by measuring the self-renewing ability of mCBSCs, using a colony-forming assay. The lower density of mCBSCs (both EP and LP) showed the formation of healthy colonies (Figure 2B), indicating the self-renewing ability in both EP- and LP-mCBSCs. Furthermore, the self-renewing ability in mCBSCs was higher than that in mMSCs (Figure 2B). We next examined the multilineage differentiation ability between mMSCs, EP-mCBSCs, and LP-mCBSCs. The mMSCs exhibited adipogenic, osteogenic, and chondrogenic differentiation, as shown in Figure 2C, while mCBSCs (EP and LP) exhibited only chondrogenic differentiation (Figure 2C). These results indicate that, among three lineages, mCBSCs have differentiation directly into the chondrogenic lineage only.

### 2.3. The Feature of Glycan Profiles in mCBSCs (EP and LP) and mMSCs

**Figure 3:**
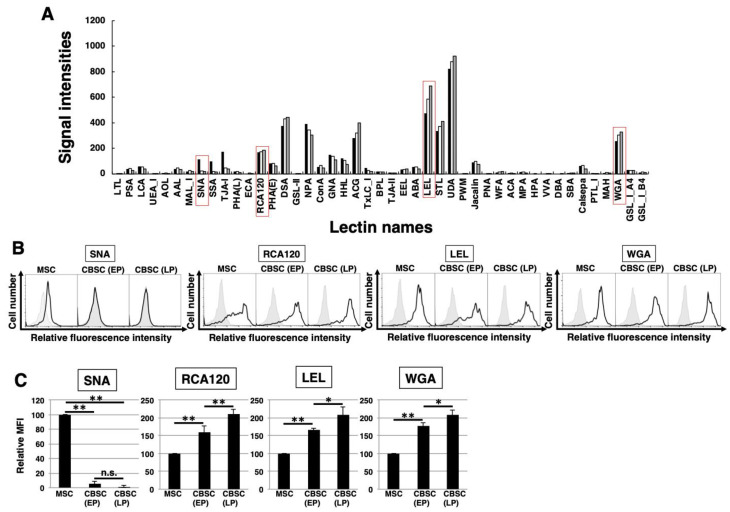
The feature of glycan profiles of mCBSCs and mMSCs. (**A**) Glycan profiles of the mMSCs, mouse cortical-bone-derived stem cells at early passage (EP-mCBSCs) and those at late passage (LP-mCBSCs) by averaged data (*n* = 10). Bar graph representation of signal intensities of 45 lectins in lectin microarray data. Closed, open, and gray bars show mMSCs, EP-mCBSCs, and LP-mCBSCs, respectively. The lectins enclosed in a red line are further experimented in (**B**) and (**C**). (**B**) FACS analysis of mMSCs, EP-mCBSCs, and LP-mCBSCs using lectins (SNA, RCA120, LEL, and WGA). Three independent experiments were performed and representative results are shown. Negative control is shown in gray. (**C**) MFIs relative to those of mMSCs (value = 100) are shown. Results are presented as means ± SD from three independent experiments. * *p* < 0.05, ** *p* < 0.01. Abbreviations: mMSC, mouse mesenchymal stem cell; mCBSC, mouse cortical-bone-derived stem cell; EP, early passage; LP, late passage; SNA, Sambucus nigra; RCA120, Ricinus communis agglutinin I; LEL, Lycopersicon esculentum; WGA, wheat germ agglutinin; MFIs, mean fluorescence intensities.

To identify the glycan profiles of the mCBSCs and mMSCs, lectin microarray analysis was performed. As a result, the signal intensities were observed in various types of lectins (Figure 3A and Table 1). Initially, the glycan profiles of both EP- and LP-mCBSCs were compared with mMSCs. The signal intensities of SNA, SSA, and TJA-I (α2-6sialic acid-binding lectins) in mMSCs were significantly higher than those in both EP- and LP-mCBSCs. The signal intensity of TxLC-I in mMSCs was slightly higher than that in both mCBSCs. In contrast, the signal intensities of RCA120 and DSA (lactosamine (Galβ1-4GlcNAc)-binding lectins), and LEL (poly lactosamine- or poly *N*-acetylglucosamine-binding lectin) in both EP- and LP-mCBSCs were higher than those in mMSCs. The signal intensity of WFA (LacdiNAc (GalNAcβ1-4GlcNAc)-binding lectin) in mCBSCs was slightly higher than that in mMSCs. Moreover, the signal intensities of MAL-I, ACG, and WGA (α2-3sialic acid-binding lectins) in mCBSCs were higher than those in mMSCs. These results suggest that there are differences in sialic acid structures and the number of lactosamine units between mMSCs and mCBSCs. 

Next, the glycan profiles were analyzed with regard to cellular aging of mCBSCs by comparing between EP and LP. The signal intensities of PSA, LCA, and AAL (α1-6fucose-binding lectins) decreased with LP-mCBSCs. The signal intensities of LEL and STL (poly *N*-acetylglucosamine-binding lectin), RCA120, and WGA increased with LP-mCBSCs. These results suggest that the glycan profile in CBSCs changes with cellular aging.

Furthermore, we performed lectin blotting and FACS analysis regarding SNA, RCA120, LEL, and WGA. Lectin blotting analysis of whole-cell lysates showed that the SNA-recognized proteins were reduced and RCA120-, LEL-, and WGA-recognized proteins were increased in the proteins marked in red (Figure S1). FACS analysis demonstrated that cell surface expression levels of SNA-binding glycan in CBSCs were significantly less compared to mMSCs (Figure 3B,C). In contrast, expression levels of RCA120-, LEL-, and WGA-binding glycans on the cell surface of CBSCs were significantly higher than those on mMSCs. Additionally, the expression was increased with passage numbers (Figure 3B,C). Taken together, these results confirmed the results of lectin microarray analysis regarding SNA, RCA120, LEL, and WGA, as described above.

### 2.4. Comparison of Glycan Profiles between mCBSCs and mMSCs Using Statistical Analysis

**Figure 4:**
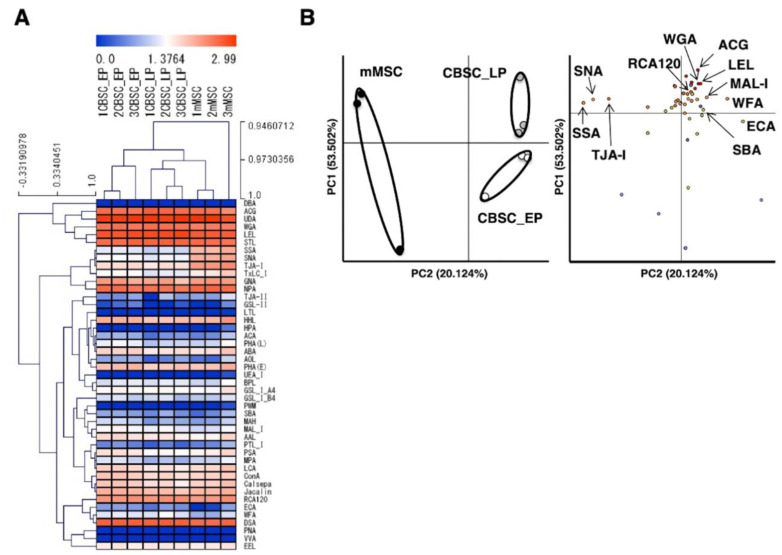
Comparison of glycan profiles between mCBSCs and mMSCs using statistical analysis. (**A**) Heat map representation of the (log10-transformed) lectin microarray data. The rows represent the lectins, and the columns represent EP-mCBSCs, LP-mCBSCs, and mMSCs with each three cells. The color scale indicates low (blue) to high (red) signal intensity. This figure shows the correlation among three types of cells by hierarchical clustering. (**B**) Biplot for principal component analysis. Left panel: cell replications; right panel: lectin replications shown as a biplot. Closed, open, and gray circles show mMSCs, EP-mCBSCs, and LP-CBSCs, respectively. Abbreviations: mMSC, mouse mesenchymal stem cell; mCBSC, mouse cortical-bone-derived stem cell; EP, early passage; LP, late passage.

To examine the features of glycans in EP- and LP-mCBSCs, and mMSCs, a hierarchical clustering and principal component analysis (PCA) were carried out with 45 lectins. Figure 4A shows the heat map of EP- and LP-mCBSCs, and mMSCs lectin microarray signals. This result indicates that the relative intensities of some lectins significantly differ by the cell types; the relative intensities of three lectins WFA, ECA (lactosamine-binding lectin), and MAL-I in EP- and LP-mCBSCs were higher than those in mMSCs. Moreover, the relative intensities of the three lectins, SNA, SSA, and TJA-I, in EP- and LP-mCBSCs were significantly lower than those in mMSCs. Thus, each EP- and LP-mCBSCs, and mMSCs showed a closeness in proximity, indicating a similarity in cell types.

By comparing lectin microarray data from EP- and LP-mCBSCs, and mMSCs, we identified the different characteristics of glycans associated with these stem cell types. As shown in Figure 4B, PC2 discriminated between mCBSCs (EP and LP) and mMSCs from the positive to the negative directions. The specific lectins of mMSCs were SSA, SNA, and TJA-I, indicating the presence of α2-6sialic acid. In contrast, the specific lectins of mCBSCs were WFA, LEL, and WGA, with a significant difference between mCBSCs and mMSCs in the positive direction on PC2. There were no significant differences in MAL-I, ECA, SBA (GalNAc-binding lectin), and RCA120 lectins; however, based on PC2, it suggested that they are characteristic lectins in mCBSCs. Moreover, EP- and LP-mCBSCs were discriminated on PC1. These results indicate that MSCs and CBSCs can be discriminated by glycan profiles, and the glycans in passaged CBSCs are varied with cellular aging.

### 2.5. The Cell Cycle Feature and Transforming Growth Factor-β1 (TGF-β1) Expression in mCBSCs

**Figure 5:**
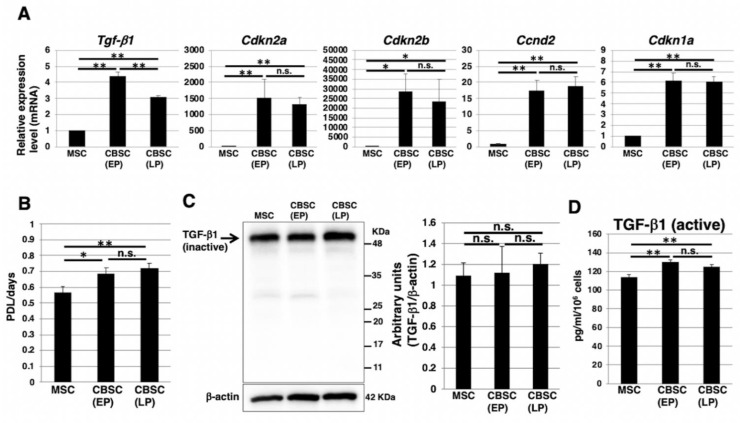
The cell cycle feature and TGF-β1 expression in mCBSCs. (**A**) Real-time PCR analysis of *Tgf-β1*, *Cdkn2a*, *Cdkn2b*, *Ccnd2,* and *Cdkn1a* in mMSCs and mCBSCs (EP and LP). The results are shown after normalization against the values obtained for mMSCs (value = 1). The values shown are the means ± SD from three independent experiments. * *p* < 0.05; ** *p* < 0.01. (**B**) Growth rate in mMSCs and mCBSCs (EP and LP) is shown by PDL/days. Data are expressed as mean ± SD from three independent experiments. * *p* < 0.05; ** *p* < 0.01. (**C**) Western blot analysis of TGF-β1 and β-actin (loading control) in mMSCs and mCBSCs (EP and LP). The blot images were cropped to highlight the β-actin bands. The histogram shows the mean densitometric analysis ± SD of TGF-β1 normalized to the loading control (β-actin). The values were obtained from three independent experiments. n.s.: not significant. (**D**) Cell culture supernatant from mMSCs and mCBSCs (EP and LP) was subjected to ELISA detection for TGF-β1 levels. The values shown are the means ± SD from triplicate measurements. ** *p* < 0.01, n.s.: not significant. Abbreviations: mMSC, mouse mesenchymal stem cell; mCBSC, mouse cortical-bone-derived stem cell; EP, early passage; LP, late passage; PDL, population doubling level.

In our previous report [14], RNAseq analysis revealed that the expression of TGF-β1 and cell-cycle-related genes was higher in mCBSCs than that in mMSCs. Real-time PCR analysis of the mCBSCs cultivated and established this time confirmed that the expression of TGF-β1 and various cell-cycle-related genes (*CDKN2A*, *CDKN2B*, and *CCND2*) were higher in both EP- and LP-mCBSCs than in mMSCs (Figure 5A). We then compared the growth rate by population doubling level (PDL)/days between mCBSCs and mMSCs. The doubling time of mCBSCs was about 15–20 h, indicating that proliferation ability of mCBSCs is relatively fast compared to mMSCs. The growth rate was significantly higher in both EP- and LP-mCBSCs than mMSCs (Figure 5B). Next, we compared protein expression levels of TGF-β1 between mCBSCs and mMSCs. Immunoblotting analysis of whole-cell lysates showed that there were no significant differences in expression levels of inactive-formed TGF-β1 between mCBSCs and mMSCs (Figure 5C). In contrast, enzyme-linked immunosorbent assay (ELISA) analysis revealed that active-formed TGF-β1 released into cell culture media was expressed higher in both EP- and LP-mCBSCs than in mMSCs (Figure 5D).

### 2.6. The Functional Properties of TGF-β1 Derived from mCBSCs

**Figure 6:**
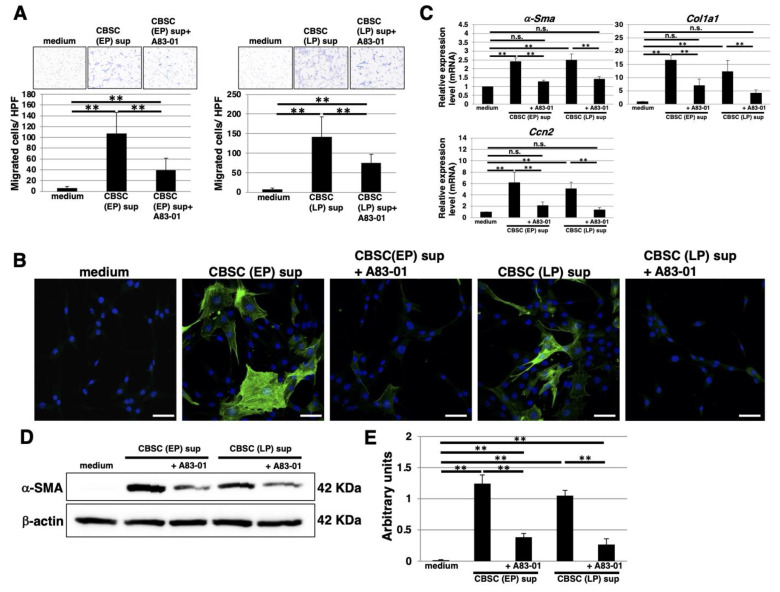
The functional properties of TGF-β1 derived from mCBSCs. (**A**) Migration assays were performed in mCBSCs (EP and LP). CBSCs growth media, mCBSCs culture media, or mCBSCs culture media with 1μM A83-01 was used for chemoattractant. Representative results from measurements of 12 fields are shown. ** *p* < 0.01. (**B**) Immunocytochemical staining was performed in NIH/3T3 cells cultured with CBSCs growth media, mCBSCs culture media, or mCBSCs culture media with 1 µM A83-01. Representative images are shown (α-SMA, *green*; DAPI, *blue*). Scale bar in the image: 50 μm. (**C**) Real-time PCR analysis of *α-Sma*, *Col1a1*, and *Ccn2* in NIH/3T3 cells cultured with CBSCs growth media, mCBSCs culture media, or mCBSCs culture media with 1 µM A83-01. The results are shown after normalization against the values obtained for NIH/3T3 cells cultured with CBSCs growth media (value = 1). The values shown are the means ± SD from three independent experiments. ** *p* < 0.01, n.s.: not significant. (**D**) Western blot analysis of α-SMA and β-actin (loading control) was performed in NIH/3T3 cells cultured with CBSCs growth media, mCBSCs culture media, or mCBSCs culture media with 1 µM A83-01. The blot images were cropped to highlight the α-SMA and β-actin bands. (**E**) The histogram shows the mean densitometric analysis ± SD of α-SMA normalized to the loading control (β-actin). The values were obtained from three independent experiments. ** *p* < 0.01. Abbreviations: mMSC, mouse mesenchymal stem cell; mCBSC, mouse cortical-bone-derived stem cell; EP, early passage; LP, late passage.

We examined the effect of TGF-β1 secreted from mCBSCs. Treatment of mCBSC-conditioned media from both EP- and LP-mCBSCs induced migration of mCBSCs. The mCBSC migration was attenuated by supplementation of A83-01, an inhibitor of TGF-β1 signaling, indicating that autocrine/paracrine TGF-β1 signaling contributes to self-migration of mCBSCs toward TGF-β1 (Figure 6A). We tested to see if TGF-β1 secreted by mCBSCs promotes the myofibroblast differentiation. It is a well-known response that TGF-β1 stimulates fibroblast differentiation into α-smooth-muscle-actin-positive (α-SMA+) myofibroblasts and contributes to the post-MI myocardial repair [29]. Treatment of mCBSC-conditioned media derived from both EP- and LP-mCBSCs greatly increased the rate of α-SMA+ myofibroblasts (Figure 6B). We also assessed the shift in fibroblast gene expression profile using a real-time PCR analysis. We found that the conditioned media treatments significantly upregulate expression of genes *α-Sma*, collagen type 1 alpha 1 chain (*Col1a1*), and cellular communication network factor 2 (*Ccn2*) that are commonly expressed in myofibroblasts (Figure 6C). To confirm the real-time PCR results, we analyzed α-SMA protein abundance using Western blot. We found that the conditioned media significantly increased α-SMA protein abundance within the fibroblasts (Figure 6D,E). The upregulation of the myofibroblast gene expressions, α-SMA protein abundance, and rate of α-SMA + cells were all attenuated by supplementing the A83-01 in the conditioned media, demonstrating that TGF-β1 secreted from mCBSCs promotes myofibroblast differentiation (Figure 6B–E). These results demonstrate that TGF-β1 released from mCBSCs contributes to differentiation of fibroblasts into myofibroblasts.

## 3. Discussion

We established mCBSCs using a protocol described previously [8]. This time, we cultured the cells for a longer period of time. The expression patterns of markers, such as c-kit (CD117), Sca-1, and β1-integrin (CD29), and lack of CD34 and CD45, were the same as those previously reported [8,14], while the expression of c-kit was about 90% in previously reported cells, but, in our case, it decreased to about 40% in EP- and about 10% in LP-mCBSCs. Expression of c-kit was considered to be affected by the culture period. In comparison with mMSCs, LP-mCBSCs cultured for a longer period than EP-mCBSCs also expressed higher levels of *Tgf-β1*, *Cdkn2a*, *Cdkn2b*, and *Ccdn2* at the mRNA level and exhibited high proliferative properties, similar to those previously reported [14]. Furthermore, self-renewal is also observed in our mCBSCs, suggesting that mCBSCs maintain stem cell properties even in long-term culture. Therefore, it is considered that mCBSCs have high utility potential. In the future, considering the effects of individual aging, it will be necessary to verify in vivo study, such as recovery of MI, including CBSC-derived from aged mice.

We found that the self-renewing ability of CBSCs is higher compared to MSCs. Previous research has shown that glycans contribute to regulation of the signaling mediated by leukemia inhibitory factor (LIF), Wnt, FGF, bone morphogenetic protein BMP, and Notch, which are required for the maintenance of stem cells [30]. It has been demonstrated that WFA-binding glycans on LIF receptorβ and gp130 regulate LIF/STAT3 signaling, which is required for self-renewal of mouse embryonic stem cells (mESCs) [22]. In this study, we found that WFA-binding glycans are specific to CBSCs compared to MSCs (Figure 4B). Further studies will be required, but we speculate that WFA-binding glycans may contribute to the self-renewal of CBSCs by regulating LIF/STAT3 signaling. Heparin/heparin sulfate (HS) proteoglycans are required for bFGF-induced dimerization and transphosphorylation of FGF receptors, the key events by which tyrosine kinase receptors initiate downstream signaling [31]. It is widely known that human embryonic stem cells require FGF signaling to sustain self-renewal and the contribution of HS to FGF signaling-mediated self-renewal is suggested [32,33,34]. bFGF supplemented in the growth medium possibly contributed to the self-renewal of CBSCs. In the future, further studies will be needed for clarification of the expression of HS and the maintenance of the undifferentiated state via HS-mediated bFGF signaling in CBSCs.

It is well documented that glycosylation of proteins is largely modified during differentiation in stem cells [18,19,20,21]. In hMSCs, the glycan profile shifts toward a higher abundance of complex *N*-glycans with more fucosylation during the differentiation into osteoblasts [21]. It is known that α2-6sialic acid is required for MSC differentiation [24,35]. In this study, we found that mCBSCs express lower levels of α2-6sialic acid on their cell surface compared to mMSCs. This lower expression of α2-6sialic acid may be an indication of specific differentiation towards chondrogenic lineage and not towards adipogenic or osteogenic differentiation. In contrast, our previous report [8] showed that implanted CBSCs can differentiate into major cardiac cell types, including cardiomyocytes, vascular smooth muscle, and endothelial cells. The present study did not find evidence that mCBSCs differentiate into those cardiac cell types. Further elucidation determining mCBSC differentiation capacity as well as the glycan profiles during those differentiations will be required. Modulation of *N*- and *O*-glycans affects the osteogenic differentiation of MSCs [23]. Identification of the lineage-specific glycans in mCBSCs may be a useful indicator for efficient differentiation of the osteogenic differentiation. Therefore, further study will be required to understand the role of glycans in the differentiation ability of mCBSCs to other lineages, including major cell types of the adult heart.

Migration of stem cells toward sites of injury is important to augment cardiac repair after injury. In this study, we showed that EP- and LP-mCBSCs released TGF-β1, which directly increased the rate of mCBSC migration. Several paracrine factors, such as chemoattractant, are released by transplanted stem cells if/when they arrive at the site of injury, leading to attracting host cells to the injury site for repair [36]. Further study should be required to clarify whether mCBSCs can migrate into an injury site via autocrine/paracrine TGF-β1 and how mCBSCs may contribute to cardiac repair after MI. In the proliferative phase of infarct healing, myofibroblasts converted from fibroblasts by growth factors, including TGF-β1, synthesize periostin and secrete large amounts of extracellular matrix proteins at the border zone [37,38]. Myofibroblasts also play important roles in the successful formation of granulation tissue and matrix remodeling in wound healing after MI [39]. Therefore, we speculate that migrated CBSCs expressing TGF-β1 may contribute to converting cardiac fibroblasts into myofibroblasts at an infarcted site. There is accumulated evidence that the adaptive immune response is involved in post-ischemic cardiac remodeling after MI. Regulatory T cells (Tregs) are known to play an important role in the resolution of inflammation and cardiac repair following MI by producing IL-10, IL-13, and TGF-β1 [40]. Some studies have suggested that TGF-β1 plays a critical role in the induction of FoxP3 expression and is a main regulator of Tregs, in vivo and in vitro [41]. Stem cells can induce Tregs via direct and indirect mechanisms. Therefore, CBSC-derived TGF-β1 may be involved in the induction of Tregs. It is known that TGF-β1 can inhibit T-lymphocyte proliferation [42] and it has been demonstrated that anti-TGF-β1 antibodies can restore T-lymphocyte proliferation [43]. The relationship between CBSC and regulation of T-lymphocyte needs to be clarified in the future, but our findings suggest that CBSCs might have a potential role in regulating immune suppression after CBSC delivery.

## 4. Materials and Methods

### 4.1. CBSC Isolation and Cell Culture

CBSC isolation and culture were performed according to a previous report [8]. CBSCs were isolated from biopsies obtained from hard bone of enhanced green fluorescent protein+ C57BL/6 mice (male, 12 weeks of age, Japan SLC inc.). The bone marrow was flushed out before taking the bone biopsies (3 mm). The bone biopsy was digested in collagenase II for an hour and passed through 100 and 40 µm. The remaining cells were plated in CBSC growth media (DMEM/F12 supplemented with 10% FBS, LIF, ITS, EGF, and bFGF) until a homogenous population of stem cells was obtained >95%. Bone-marrow-derived mMSCs were obtained from RIKEN BioResource Research Center and cultured in CBSC growth media. NIH/3T3 mouse fibroblast cells were obtained from JCRB Cell Bank and cultured in CBSC growth media. SA-β-Gal activity was assayed using a senescence detection kit (BioVision Inc., Milpitas, CA, USA), as described in our previous reports [44]. Briefly, cells were washed twice with PBS, exposed to fixation solution for 10 min, and then incubated overnight in freshly prepared staining solution. For induction of stress-induced premature senescence, cells were treated with 400 µM H_2_O_2_ for 2 h. After washing with PBS, cells were re-cultured in fresh medium for 72 h. 

### 4.2. Multilineage Differentiation

Adipogenic, chondrogenic, and osteogenic differentiations were induced using a differentiation kit (Stem Cell Technologies, Vancouver, Canada) according to the manufacturer’s protocol. For evaluation of adipogenic differentiation, cells were fixed with 10% formalin and neutral lipids were detected with Oil Red O Stain (Sigma-Aldrich, St. Louis, MO, USA). Images were acquired with a phase contrast light microscope (Leica Microsystems, Wetzlar, Germany). For evaluation of chondrogenic differentiation, pellets were fixed with formalin, embedded in paraffin, and were sectionalized. Sections were stained with Alcian blue. Images were acquired with Mantra, multi-spectral microscopy (PerkinElmer, Waltham, MA, USA). For evaluation of osteogenic differentiation, cells were fixed with 10% formalin and stained with Alizarin Red solution (PG Research, Tokyo, Japan). Images were acquired with a phase contrast light microscope.

### 4.3. Self-renewal Assay

For evaluation of colony forming ability, the cells were harvested and the dissociated single cells were seeded in 6-well plates at low density (250, 500, 1000 cells per well) and cultured for 7 days. The plates were then washed with phosphate-buffered saline (PBS) and fixed with 10% formalin for 10 min, followed by staining with a Diff-Quick staining kit (Polysciences, Inc., Warrington, PA, USA) for 30 min. The plates were then washed with PBS and images of each well were captured.

### 4.4. FACS Analysis

Cells were harvested and these dissociated single cells were incubated with fluorescein-conjugated primary antibodies or negative isotype control in FACS buffer (0.5% BSA and 0.1% sodium azide in PBS) for 30 min on ice. For detection of lineage cocktail, after staining with biotin-conjugated antibody, cells were stained with anti-biotin-APC-conjugated antibody. After washing, cell sorting and analysis were performed using an FACS Aria Cell Sorter (Becton Dickinson, Franklin Lakes, NJ, USA). All used antibodies are listed in Table S1. For lectin staining, biotin-conjugated Sambucus nigra (SNA) (EY Laboratories, San Mateo, CA, USA), biotin-conjugated Ricinus communis agglutinin I (RCA120) (Vector Laboratories, Peterborough, UK), biotin-conjugated Lycopersicon esculentum (LEL) (Vector Laboratories), biotin-conjugated wheat germ agglutinin (WGA) (J-Oil Mills, Tokyo, Japan), and TexasRed-conjugated streptavidin (Vector Laboratories) were used. Mean fluorescence intensities (MFIs) were calculated by subtracting the intensities of the negative controls.

### 4.5. Immunoblotting

Cells were lysed with lysis buffer (50 mM Tris HCl pH 7.4, 150 mM NaCl, and 1% (*v/v*) Triton™ X-100) containing protease and phosphatase inhibitor cocktails (Roche, Indianapolis, IN, USA). Total lysates were separated by SDS-PAGE using an 8% gel and then transferred onto PVDF membranes (Merck Millipore, Billerica, MA, USA). After blocking, the membranes were incubated with the following primary antibodies: monoclonal mouse anti-Nanog (dilution 1:1000; Santa Cruz Biotechnology, Dallas, TX, USA), polyclonal rabbit anti-Sox2 (dilution 1:1000; Merck Millipore), monoclonal rabbit anti-TGF-β1 (dilution 1:1000; Abcam, Cambridge, UK), monoclonal mouse anti-α-SMA (dilution 1:1000; Abcam), and monoclonal mouse anti-β-actin (dilution 1:10,000; Sigma-Aldrich). For lectin blot, the membranes were incubated with biotin-conjugated lectins: biotin-conjugated SNA, biotin-conjugated RCA120, biotin-conjugated LEL, and biotin-conjugated WGA. The membranes were then incubated with the appropriate peroxidase-conjugated secondary antibodies (dilution 1:30,000; Cell Signaling Technology), or peroxidase-conjugated streptavidin (Jackson ImmunoResearch Labs, West Grorve, PA, USA) for biotin-conjugated lectin, washed, and developed with ECL™ Prime reagents (GE Healthcare, Piscataway, NJ, USA).

### 4.6. Immunocytostaining

Cells were fixed with 4% (*w/v*) paraformaldehyde and washed. Next, cells were permeabilized and blocked with PBS containing 0.2% (*v/v*) Triton™ X-100, 1% (*w/v*) BSA, and 5% (*v/v*) normal goat serum. After washing, cells were incubated with an anti-α-SMA antibody (dilution 1:100) at 4 °C overnight. After washing, cells were stained with an Alexa Fluor^®^ 488-conjugated secondary antibody (dilution 1:400; Molecular Probes) and then counterstained with DAPI. Immunofluorescence images were taken with a confocal laser scanning microscope (Leica Microsystems).

### 4.7. Lectin Array Analysis

Lectin microarray analysis of hydrophobic protein extracts was performed as previously described [45,46]. Briefly, 0.2 μg of total proteins, including glycoproteins, was labeled with Cy3 mono-reactive dye (GE Healthcare, Buckinghamshire, UK) in PBS at r.t. for 1 h. The excess dye was removed using a spin-type column loaded with Sephadex G-25 fine matrix (GE Healthcare) and the collected Cy3-labeled glycoprotein solution was diluted to 2 μg/mL with probing buffer (Tris-buffered saline containing 1% Triton X-100, 1 mM CaCl_2_, and 1 mM MnCl_2_, pH 7.4). The glycoprotein solution (0.5 µg/mL) was applied to a LecChip (ver.1.0; GlycoTechnica, Yokohama, Japan) containing 45 lectins (Table S2). After incubating at 4 °C for approximately 17 h, the reaction solution was discarded. The LecChip was washed three times with probing buffer before scanning using the evanescent-field fluorescence scanner, GlycoStationTM Reader 1200 (GlycoTechnica). Each sample was measured in triplicate or quadruplicate. Data were analyzed using GlycoStationTM Tools Signal Capture 1.0 and GlycoStationTM Tools Pro 1.0 (GlycoTechnica). For accurate analysis, the data were normalized by the mean of 45 lectin signals on a well for each array, i.e., average-normalization [46].

### 4.8. ELISA Analysis

The cell culture supernatants were analyzed for active mouse TGF-β1 levels by ELISA kit (Proteintech, Rosemont, IL, USA), following the manufacturer’s protocol. The productivity rate was calculated from the concentration of TGF-β1 with respect to the cell number.

### 4.9. Migration Assay

Cell culture inserts (8-μm pore size and 6 mm in diameter) were used according to the manufacturer’s instructions. CBSC growth media, mCBSC culture media, or mCBSC culture media with 1 μM A83-01 (FUJIFILM Wako Chemicals, Osaka, Japan), a potent inhibitor of TGF-β type I receptor signaling, was placed in the lower chamber. The cells were plated at 1 × 10^5^ cells/500 μL onto the upper component of the inserts and, after 16 h, the number of cells that had migrated through the membrane to the lower surface of the filter were fixed and stained with a Diff-Quick staining kit. The images were taken using Mantra, multi-spectral microscopy, and then images were loaded into inForm software ver. 2.4 (PerkinElmer) to count the number of migrated or invaded cells in 12 random fields with a 20× magnification objective.

### 4.10. Real-Time PCR

Total RNA was isolated from mMSCs and mCBSCs using an RNeasy plus mini-kit (QIAGEN, Hilden, Germany) and, subsequently, reverse-transcribed using a ReverTra Ace^®^ qPCR RT Kit (Toyobo, Osaka, Japan). Real-time PCR was performed by using a Power Sybr^®^ Green kit (Applied Biosystems, Foster City, CA, USA) and a StepOnePlus™ real-time PCR system (Applied Biosystems). Primer sets for real-time PCR were as follows: *Tgf-β1*: 5′-CTGGGCACCATCCATGACA-3′(F), 5′-GCCGCACACAGCAGTTCTT-3′(R); *Cdkn2a*: 5′-CGCAGGTTCTTGGTCACTGT-3′(F), 5′-TGTTCACGAAAGCCAGAGCG-3′(R); *Cdkn2b*: 5′-CCCTGCCACCCTTACCAGA-3′(F), 5′-CAGATACCTCGCAATGTCACG-3′(R); *Ccnd2*: 5′-AGGAGAAGCTGTCCCTGATCC-3′(F), 5′-AGTTGCAATCATCGACGGC-3′(R); *Col1a1*: 5′-GTATGCTTGATCTGTATCTG-3′(F), 5′- CGACTCCTACATCTTCTG-3′(R); *Ccn2*: 5′-CTGCGAGGAGTGGGTGTG-3′(F), 5′- ATGTGTCTTCCAGTCGGTAGG-3′(R); *Gapdh*: 5′-CCAATGTGTCCGTCGTGGATCT-3′(F), 5′-GTTGAAGTCGCAGGAGACAACC-3′(R). *Gapdh* was used as an internal control to normalize target gene transcripts.

### 4.11. Statistical analysis

Western blot images were analyzed by using ImageJ software (National Institutes of Health). Values were expressed as means ± standard deviation (SD) from three independent experiments. One-way ANOVA was performed for comparing multiple groups. Lectin microarray data from triplicate or quadruplicate analysis were performed and displayed using TIGR MultiExperiment Viewer (http://www.tm4.org/mev.html, accessed on 14 October 2021). The values were calculated as log10. Data were also evaluated by hierarchical clustering and PCA with pair-wise comparisons (http://lgsun.grc.nia.nih.gov/ANOVA/, accessed on 14 October 2021; false discovery rate < 0.05). The lectin microarray data were confirmed using the *t*-test, performed on an Excel software to determine statistical significance (*p* < 0.05). The signal intensities of three cells in each type of cell were used for the *t*-test.

## 5. Conclusions

In this study, we examined the stem-cell-like characteristics, the cell surface glycan profile, and the functional cellular features in mCBSCs compared with bone-marrow-derived mMSCs. The stem cell feature, such as self-renewing ability, in mCBSCs was higher than that in mMSCs. In contrast, the differentiation ability of mCBSCs was limited in the chondrogenic lineage among three types of cell (adipocyte, osteoblast, chondrocyte), while mMSCs can differentiate into all three types of cell. Further study will be required to clarify whether mCBSCs have higher chondrogenicity than other MSCs, such as synovium-derived MSCs, which have superior chondrogenicity. The cell surface glycan profiles by lectin array analysis and FACS analysis revealed that α2-6sialic acid is expressed at very weak levels on the cell surface of mCBSCs compared with that on mMSCs, and that RCA120-binding glycan (lactosamine (Galβ1-4GlcNAc)-), LEL-binding glycan (poly lactosamine- or poly *N*-acetylglucosamine-), and WGA-binding glycan (α2-3sialic acid on both *N*- and *O*-glycans) are particularly expressed at higher levels than those on mMSCs. Furthermore, these glycans expressed were increased with long-term cell culture, so-called in vitro cellular aging. Moreover, the clarification of in vitro differentiation of mCBSCs into major cardiac cell types, including cardiomyocytes, vascular smooth muscle, and endothelial cells, with the glycan profiles will be required. Those characterizations in mCBSCs may be useful for the development of the efficient delivery of disease-specific differentiated cells, which may be prepared by glycan modification. We further found that TGF-β1 released from mCBSCs contributed to the self-migration of mCBSCs and the activation of fibroblasts. These results suggest that implanted mCBSCs may migrate toward the injury site by autocrine/paracrine TGF-β1 and activated fibroblasts by TGF-β1 of mCBSCs may contribute to cardiac repair during the post-MI wound healing processes. Additional studies are required to clarify whether mCBSCs can migrate into an injury site via autocrine/paracrine TGF- β1, leading to cardiac repair. 

## Figures and Tables

**Table 1:** The signal intensities of each cell type in lectin microarray data.

Lectin Name	mMSC-1	mMSC-2	mMSC-3	CBSC (EP)-1	CBSC (EP)-2	CBSC (EP)-3	CBSC (LP)-1	CBSC (LP)-2	CBSC (LP)-3
LTL^*2^	0.1	0.1	1.3	0.2	0.1	0.6	0.0	0.0	0.1
PSA^*2^	29.6	33.1	50.5	41.3	45.4	36.8	23.5	28.5	23.8
LCA^*2^	46.2	49.9	74.4	56.6	59.2	50.1	35.7	41.8	34.2
UEA_I	0.1	0.0	3.5	0.0	0.1	0.5	0.0	0.1	0.3
AOL^*2^	2.8	2.5	15.7	5.8	6.5	7.2	2.0	3.5	6.1
AAL^*2^	28.6	28.9	54.6	48.2	52.4	38.6	36.4	40.4	31.4
MAL_I^*2^	13.1	14.6	21.9	27.2	30.6	21.1	18.8	22.1	16.6
SNA^*1, *2^	105.2	104.2	129.8	25.2	23.2	22.0	16.7	20.5	20.7
SSA^*1, *2^	89.2	84.6	112.9	17.7	18.2	16.3	11.1	15.2	12.7
TJA-I^*1, *2^	153.2	160.4	197.7	45.0	45.0	47.5	34.8	44.8	40.9
PHA(L)^*2^	11.8	12.5	27.4	18.9	21.3	19.0	9.0	12.7	12.7
ECA^*2^	1.1	1.4	5.7	5.3	5.6	5.2	2.9	5.3	4.4
RCA120	148.3	151.8	194.2	162.8	167.2	187.2	164.3	191.9	192.9
PHA(E)^*2^	66.1	70.0	107.9	74.1	80.4	94.3	54.3	60.7	74.6
DSA^*1^	354.7	354.1	405.5	426.4	424.0	443.8	423.0	454.3	450.3
GSL-II^*2^	0.5	1.1	4.0	1.7	2.4	3.7	0.1	0.8	2.0
NPA^*1^	400.6	413.7	369.2	373.9	372.4	302.9	326.5	313.9	282.0
ConA^*1, *2^	48.8	53.1	58.7	69.1	71.0	58.7	45.4	51.0	41.2
GNA	144.5	162.0	145.7	152.2	164.2	105.9	122.0	147.0	72.1
HHL^*2^	90.0	89.5	165.5	111.7	102.1	103.8	68.3	67.8	81.0
ACG^*2^	341.5	331.2	193.7	349.2	324.7	298.2	456.7	396.0	355.6
TxLC_I^*1, *2^	33.2	36.7	62.0	24.1	26.8	26.1	16.1	18.2	17.8
BPL	12.6	11.9	23.9	14.0	17.0	18.1	14.9	17.8	17.0
TJA-II	5.9	5.5	17.4	4.2	4.3	6.1	1.0	9.5	5.3
EEL	33.0	35.1	39.7	34.3	38.5	32.3	35.8	37.0	37.3
ABA^*2^	37.9	42.8	71.0	54.5	55.4	57.3	35.5	39.7	44.0
LEL^*1, *2^	486.8	474.2	469.9	577.2	540.0	625.4	645.6	656.2	747.8
STL^*2^	376.3	336.5	305.0	349.0	342.0	409.7	402.3	424.4	405.9
UDA	984.6	965.7	600.8	854.2	838.9	925.6	958.4	804.8	987.9
PWM	0.1	0.0	1.8	0.7	0.3	1.4	0.2	0.9	0.3
Jacalin^*2^	85.1	85.3	99.3	99.7	103.6	93.8	78.2	81.8	63.5
PNA	0.0	0.0	0.3	0.1	0.0	0.0	0.0	0.0	0.2
WFA^*1^	7.1	6.7	15.0	16.0	18.0	16.1	15.3	23.7	15.6
ACA^*2^	3.4	4.0	12.7	6.8	7.5	6.5	2.6	4.6	4.9
MPA^*2^	11.4	12.0	20.0	15.6	16.1	12.1	7.1	9.4	6.8
HPA^*2^	0.0	0.0	3.3	0.3	0.6	0.8	0.0	0.0	0.0
VVA	0.0	0.0	0.1	0.0	0.0	0.0	0.0	0.0	0.1
DBA	0.0	0.0	0.0	0.0	0.0	0.0	0.0	0.0	0.0
SBA	2.5	3.8	8.8	6.1	6.4	5.4	4.2	7.1	4.4
Calsepa^*2^	54.3	56.4	75.3	69.4	72.1	62.2	42.7	48.4	32.3
PTL_I	3.4	3.8	7.9	3.5	4.7	3.4	2.9	3.6	2.2
MAH^*2^	5.1	5.6	11.9	13.6	13.9	11.0	4.6	7.7	6.3
WGA^*1^	247.6	263.2	256.9	304.0	331.3	286.0	341.9	341.1	311.2
GSL_I_A4	23.6	21.8	41.6	26.3	30.8	27.5	26.8	29.2	26.6
GSL_I_B4	10.0	10.2	16.1	13.9	16.2	10.5	12.7	16.2	7.8

The data were averaged after normalization (sample 1 and 2, *n* = 3; sample 3, *n* = 4). *T*-test was performed with signal intensities before average (*n* = 10). *1 There was the significant difference between mCBSCs (both EP and LP) and mMSCs. *2 There was the significant difference between EP-mCBSCs and LP-mCBSCs.

## Data Availability

The data presented in this study are available on request from the corresponding author. The data are not publicly available due to privacy.

## Supplementary Materials

The following are available online at https://www.mdpi.com/article/10.3390/ijms222111849/s1, Figure S1: Lectin blotting analysis, Table S1: Antibody list, Table S2: List of lectins for microarray.

## Abbreviations

CVDs: cardiovascular diseases; MI: myocardial infarction; SSCs: somatic stem cells; MSCs: mesenchymal stem cells; mCBSCs: mouse cortical-bone–derived stem cells; hMSCs: human MSCs; mMSCs: mouse mesenchymal stem cells; FACS: fluorescence-activated cell sorting; EP: early passage; LP: late passage; SA-β-Gal: senescence-associated β-galactosidase; MFIs: mean fluorescence intensities; SNA: Sambucus nigra; RCA120: Ricinus communis agglutinin I; LEL: Lycopersicon esculentum; WGA: wheat germ agglutinin; PCA: principal component analysis; PDL: population doubling level; ELISA: enzyme-linked immunosorbent assay; α-SMA: α-smooth muscle actin; TGF: transforming growth factor; LIF: leukemia inhibitory factor; mESCs: mouse embryonic stem cells; HS: heparin/heparin sulfate; SD: standard deviation; PBS: phosphate-buffered saline.
