# Supplementary material for: Cell Surface and Functional Features of Cortical Bone Stem Cells
Source: Int J Mol Sci. 2021 Oct 31;22(21):11849. doi: 10.3390/ijms222111849 (PMC8584423; doi:10.3390/ijms222111849)
Supplement: Supplementary file 1 [file ijms-22-11849-s001.zip › ijms-1443022-supplementary.pdf]

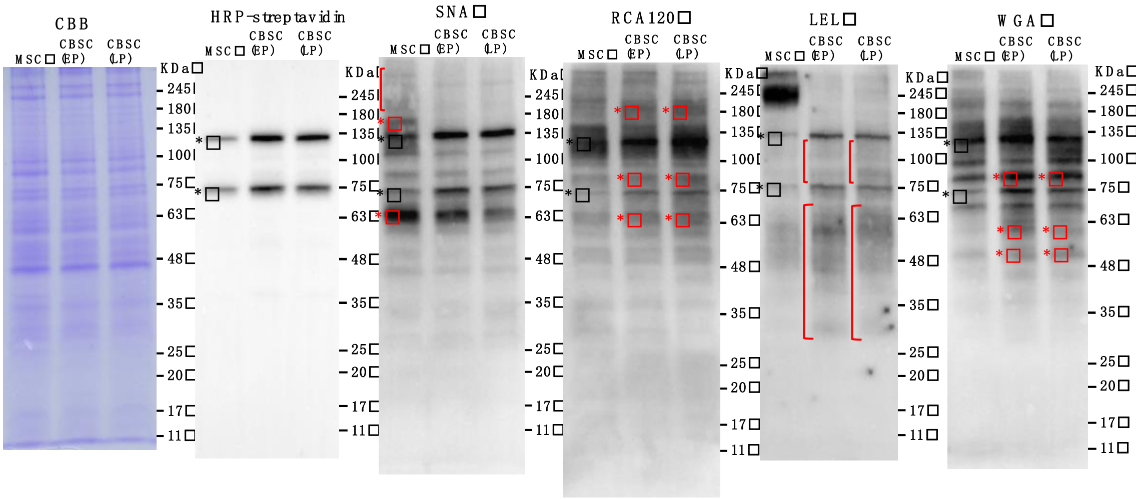

Figure S1. Lectin blotting analysis. Whole cell lysates from mMSCs, EP-CBSCs and LP-CBSCs, were analyzed by lectin blotting using lectins (SNA, RCA120, LEL, and WGA). Equal amounts of cell lysates were loaded. CBB staining showed that the loaded proteins were almost all equal. Black asterisk show non-specific binding proteins by HRP-streptavidin. Red asterisk and red range show decreased or increased glycan-conjugated proteins in mCBSCs compared with mMSCs. Abbreviations: mMSC, mouse mesenchymal stem cell; mCBSC, mouse cortical bone derived stem cell; EP, early passage; LP, late passage; SNA, *Sambucus nigra*; RCA120, *Ricinus communis agglutinin I*; LEL, *Lycopersicon esculentum*; WGA, *Wheat germ agglutinin*; CBB, coomassie brilliant blue.

Table S1 Antibody list

Marker	Primary Antibody	Negative Isotype Control
Sca-1	Rat IgG2a anti-Sca-1-APC (Miltenyi Biotec; Cologne, Germany)	Rat IgG2a-APC (Miltenyi Biotec; Cologne, Germany)
CD29	Hamster IgG anti-CD29-APC (eBiosciences; San Diego, CA)	Hamster IgG-APC (eBiosciences; San Diego, CA)
CD34	Rat IgG2a anti-CD34-APC (Novus Biologicals; Centennial, CO)	Rat IgG2a-APC (Miltenyi Biotec; Cologne, Germany)
CD45	Anti-CD45-APC (Miltenyi Biotec; Cologne, Germany)	Isotype control-APC (Miltenyi Biotec; Cologne, Germany)
C-kit	1st goat IgG anti-SCF-R (R&D Systems; Minneapolis, MN) 2nd anti-Goat IgG-Alexa Fluor 647 (Abcam, Cambridge, UK)	2nd anti-Goat IgG-Alexa Fluor 647 only (Abcam, Cambridge, UK)
Lineage Cocktail (Lin)	1st Biotin-Conjugated Lineage Cocktail (Miltenyi Biotec; Cologne, Germany) 2nd anti-Biotin-APC (Miltenyi Biotec; Cologne, Germany)	2nd anti-Biotin-APC only (Miltenyi Biotec; Cologne, Germany)

Table S2 List of lectins for microarray

Abbreviation	Lectin name	Oligosaccharide binding specificity [*1]
LTL	*Lotus tetragonolobus*	Fuca1-3(Galb1-4)GlcNAc, Fuca1-2Galb1-4GlcNAc
PSA	*Pisum sativum*	Fuca1-6GlcNAc, a-Man
LCA	*Lens culinaris*	Fuca1-6GlcNAc, a-Man
UEA-I	*Ulex europaeus*	Fuca1-2Galb1-4GlcNAc
AOL	*Aspergillus oryzae*	Fuca1-6GlcNAc, Fuca1-2Galb1-4GlcNAc
AAL	*Aleuria aurantia*	Fuca1-3(Galb1-4)GlcNAc, Fuca1-6GlcNAc
MAL-I	*Maackia amurensis*	Siaa2-3Galb1-4GlcNAc
SNA	*Sambucus nigra*	Siaa2-6Gal/GalcNAc
SSA	*Sambucus sieboldiana*	Siaa2-6Gal/GalcNAc
TJA-I	*Trichosanthes japonica*	Siaa2-6Gal/GalcNAc
PHA(L)	*Phaseolus vulgaris*	tri/tetra-antennary complex-type *N*-glycan
ECA	*Erythrina cristagalli*	Galb1-4GlcNAc
RCA120	*Ricinus communis*	Galb1-4GlcNAc
PHA(E)	*Phaseolus vulgaris*	bi-antennary complex-type *N*-glycan, bisecting GlcNAc
DSA	*Datura stramonium*	Galb1-4GlcNAc, $(GlcNAc)_n$
GSL-II	*Griffonia simplicifolia*	agalactosylated tri/tetra antennary glycans, GlcNAc
NPA	*Narcissus pseudonarcissus*	Mana1-6Man
ConA	*Canavalia ensiformis*	Mana1-6(Mana1-3)Man
GNA	*Galanthus nivalis*	Mana1-3Man
HHL	*Hippeastrum hybrid*	Mana1-3Man, Mana1-6Man
ACG	*Agrocybe cylindracea*	Galb1-3Gal, Siaa2-3Galb1-4Glc
TxLCI	*Tulipa gesneriana*	Mana1-3(Mana1-6)Man, bi-antennary complex-type *N*-glycan
BPL	*Bauhinia purpurea alba*	Galb1-3GalNAc
TJA-II	*Trichosanthes japonica*	Fuca1-2Galb
EEL	*Euonymus europaeus*	Gala1-3Galb1-4GlcNAc, Fuca1-2Galb1-3GlcNAc
ABA	*Agarics bisporus*	Galb1-3GalNAc
LEL	*Solanum lycopersicum*	$(GlcNAcb1\text{-}4)_n$, $(Galb1\text{-}4GlcNAc)_n$
STL	*Solanum tuberosum*	$(GlcNAcb1\text{-}4)_n$
UDA	*Urtica dioica*	$(GlcNAcb1\text{-}4)_n$, High-Man
PWM	*Phytolacca americana*	$(GlcNAcb1\text{-}4)_n$
Jacalin	*Artocarpus integrifolia*	Galb1-3GalNAc, GalNAca, GlcNAcb1-3GalcNAc, Mana1-6(Mana1-3)Man
PNA	*Arachis hypogaea*	Galb1-3GalNAc
WFA	*Wisteria floribunda*	GalNAcb1-4GlcNAc, Galb1-3GalNAc
ACA	*Amaranthus caudatus*	Galb1-3GalNAc, Siaa2-3Galb1-3GalNAc
MPA	*Maclura pomifera*	GalNAca, Galb1-3GalNAc
HPA	*Helix pomatia*	GalNAca
VVA	*Vicia villosa*	GalNAcb1-4Gal, GalNAcb1-3Gal, GalNAca
DBA	*Dolichos biflorus*	GalNAca1-3(Fuca1-2)Galb, GalNAca1-3GalNAc
SBA	*Glycine max*	Tarminal GalNAc
Calsepa	*Calystegia sepium*	Galactosylated bi-antennary complex-type *N*-glycan with bisecting GlcNAc, High-Man
PTL-I	*Psophocarpus tetragonolobus*	GalNAca, Gala1-3(Fuca1-2)Galb
MAH	*Maackia amurensis*	Siaa2-3Galb1-3GalNAc
WGA	*Triticum vulgaris*	$(GlcNAcb1\text{-}4)_n$, Hybrid type *N*-glycan with bisecting GlcNAc
GSL-I-A_4	*Griffonia simplicifolia*	GalNAca
GSL-I-B_4	*Griffonia simplicifolia*	Gala

Each lectin is shown as the abbreviation, the lectin name and the main specificity on LecChip (ver. 1.0).
[*1] This was modified of GlycoTechnica Ltd. web list (https://www.glycotechnica.com/en/chip/).
